# Case reports as early safety signals: learning from the COVID-19 vaccination campaign

**DOI:** 10.3389/fmed.2023.1348376

**Published:** 2024-01-09

**Authors:** Michel Goldman, Rebecca E. Chandler

**Affiliations:** ^1^I3h Institute, Université libre de Bruxelles, Brussels, Belgium; ^2^Coalition for Epidemic Preparedness Innovations, London, United Kingdom

**Keywords:** safety, vaccine, adjuvant, COVID-19, case report, pharmacovigilance

## Introduction

The development of anti-SARS-CoV-2 vaccines will stay as a landmark in the history of modern medicine, both as a major scientific achievement and an unprecedented public health success. The rapid deployment of vaccines was authorized after their overall efficacy and safety was established in randomized clinical trials. As expected, very rare adverse events were not apparent in these trials and could only be identified after the launch of large immunization programs. Indeed, since the beginning of the COVID-19 vaccination campaign, several case reports raised suspicion about rare but severe adverse events which were not identified in pre-authorization trials. While thrombotic thrombocytopenia and myocarditis were rapidly recognized as rare adverse events, there are still uncertainties about other allegations derived from case reports (1, 2). These uncertainties are important drivers of vaccine hesitancy. It is therefore important to consider how possible risk of severe adverse events suggested by case reports can best be addressed.

## Limitations of current pharmacovigilance systems

During the COVID-19 vaccination campaign, some rare adverse events could only be identified after a careful in-depth analysis of a few clinical cases. Thus, vaccine-induced immune thrombotic thrombocytopenia, a very rare but severe adverse event of adenoviral vector-based vaccines, was primarily identified by two small groups of physicians and scientists who were not pharmacovigilance experts. Indeed, the European Medicines Agency (EMA) had to revise its initial statement denying any relationship between vaccination and thrombotic events after Greinacher et al. identified a small number of atypical cases characterized by unusual sites of thrombosis, thrombocytopenia, and circulating anti-PF4 antibodies (3, 4). Likewise, it is a careful analysis of 6 case reports that led EMA to advise against the use of these vaccines in people with history of capillary leak syndrome (https://bityl.co/LtPW).

These stories remind us that passive pharmacovigilance based on existing safety monitoring systems has intrinsic limitations preventing the early identification of certain rare events (5). First and foremost, the definition of adverse events is mostly based on clinical features and does not include biological parameters which might be key for their full characterization. Consequently, the disproportionality analyses comparing their incidence in vaccinated vs. unvaccinated populations might be flawed as illustrated above for thrombotic events following adenoviral vector-based vaccination. Furthermore, passive pharmacovigilance depends on voluntary reporting by healthcare providers or patients. During the COVID-19 vaccination campaign, several unexpected events were not reported because they were considered as coincidental. Initially, in-depth investigation of adverse events linked to the new technologies underlying COVID-19 was not prioritized by research funding agencies, since even if a causal relationship would be established it would not modify the clearly favorable benefit-risk balance in the context of a major public health need. Furthermore, the few scientists engaged in such research were often considered to fuel vaccine hesitancy or even to support anti-vaccine movements. Consequently, several side effects suggested by case reports were only investigated superficially using retrospective disproportionality analyses. There have been few exceptions such as the study from the Norwegian Institute of Public Health which documented that mRNA COVID-19 vaccination increases the risk of vaginal bleeding in non-menstruating women (6). The strength of this large study based on self-reports by patients is that it was initiated at the very beginning of the vaccination campaign. Unfortunately, other suspected side effects did not benefit from careful epidemiological studies although they can have a major impact on the quality of life of patients, tinnitus being one example among others (7).

## The need for mechanistic studies

The myocardial and pericardial damages occasionally induced by COVID-19 mRNA vaccines incentivized several studies aiming at clarifying the respective roles of the Spike protein, vaccine-induced inflammation, and genetic predisposing factors on which the COVID-19 Human Genetic Effort consortium is focusing (8). Case reports of monozygotic twins who both developed myocarditis after receiving mRNA vaccine shots (9) provide a strong rationale for the latter study. As a matter of fact, there is a body of evidence that genetic variants contribute to interindividual differences in the propension to develop vaccine side effects. This previously led to propose *adversomics* as a new concept of vaccine safety based on the identification of individuals at genetic risk of adverse events (10). Besides germline gene variants, acquired somatic mutations should be considered as well, especially those occurring in the context of clonal hematopoiesis (11).

Although genetic factors are essential to consider, additional pathogenetic mechanisms are important to be studied simultaneously, taking advantage of system biology tools. Such a global approach will be implemented by the International Network of Special Immunization Services (INSIS), a large partnership which includes the Brighton Collaboration, a pioneer in vaccine safety science (https://insisvaccine.org).

Mechanistic studies should not be restricted to the study of myocarditis and thrombotic thrombocytopenia. Indeed, even though additional rare severe adverse events might not affect the overall risk-benefit balance of vaccines, it is important to identify individuals at risk and define the best way to manage them. Among the multiple allegations supported by case reports (1, 2), we suggest prioritizing the investigation of events of inflammatory nature. Inflammation mediated by cytokines is indeed the cornerstone of vaccine reactogenicity resulting in loco-regional or systemic symptoms. Regarding local reactions at the site of injection, they might occasionally be severe enough to mimic cellulitis caused by an infectious agent; a condition denominated the “COVID-19 arm” (12). Reactive lymphadenopathy affecting lymph nodes draining the injection site should also be mentioned as it might mimic metastasis in oncologic patients (13). It is initiated by a strong germinal center reaction involving B cells and follicular helper T cells (14). The adjuvant activity of the ionizable lipid component of lipid nanoparticles appears to play a key role in the induction of these lymphocyte responses (15). Although most of these responses are specific for the vaccine antigen, a strong inflammatory environment could also stimulate unrelated memory T cells in an antigen-independent manner (16). This possibility should be considered as a mechanism of adverse reactions involving quiescent malignant or autoreactive lymphocytes. Hemophagocytic lymphohistiocytosis which has been reported after COVID-19 vaccination (17) is an hyperinflammatory syndrome which deserves special attention as genetic factors predisposing to this condition have been identified (18). Unfortunately, search for genetic factors was only performed in very few cases of post-vaccine hemophagocytic lymphohistiocytosis. Interestingly, two predisposing genetic mutations were identified in one of those cases (17).

The rarity of severe adverse events of inflammatory nature is not surprising since interindividual differences in vaccine-induced inflammatory responses have been well-documented in a study comparing reactogenicity and muscle PET/CT imaging after injection of different adjuvanted vaccines in healthy volunteers (19).

## Concluding remarks

While mRNA and adenoviral vector-based vaccines are currently considered for several indications in infectious diseases and oncology, and lipid nanoparticles are increasingly used as delivery vehicles for nucleic acid therapeutics (20), multidisciplinary research on the factors that might precipitate adverse events in rare individuals should be prioritized and targeted active surveillance programs should be planned accordingly. This might help to protect individuals at risk, optimize products formulation, and overall enhance public confidence in pharmaceutical innovation. Let's remember Marie Skłodowska-Curie' saying: “Nothing in life is to be feared, it is only to be understood. Now is the time to understand more so that we may fear less.”

## Author contributions

MG: Conceptualization, Writing—original draft. RC: Conceptualization, Writing—review & editing.

## References

[1] Mohseni Afshar Z Tavakoli Pirzaman A Liang JJ Sharma A Pirzadeh M Babazadeh A. Do we miss rare adverse events induced by COVID-19 vaccination? Front Med. (2022) 9:933914. 10.3389/fmed.2022.933914 36300183 PMC9589063

[2] Yaamika H Muralidas D Elumalai K. Review of adverse events associated with COVID-19 vaccines, highlighting their frequencies and reported cases. J Taibah Univ Med Sci. (2023) 18:1646–61. 10.1016/j.jtumed.2023.08.004 37732332 PMC10507236

[3] Greinacher A Thiele T Warkentin TE Weisser K Kyrle PA Eichinger S. Thrombotic thrombocytopenia after ChAdOx1 nCov-19 vaccination. N Engl J Med. (2021) 384:2092–101. 10.1056/nejmoa2104840 33835769 PMC8095372

[4] Schultz NH Sørvoll IH Michelsen AE Munthe LA Lund-Johansen F Ahlen MT. Thrombosis and thrombocytopenia after ChAdOx1 nCoV-19 vaccination. N Engl J Med. (2021) 2021:NEJMoa2104882. 10.1056/NEJMoa2104882 33835768 PMC8112568

[5] Black SB Chandler RE Edwards KM Sturkenboom MCJM. Assessing vaccine safety during a pandemic: recent experience and lessons learned for the future. Vaccine. (2023) 41:3790–5. 10.1016/j.vaccine.2023.04.055 37198019 PMC10184950

[6] Blix K Laake I Juvet L Robertson AH Caspersen IH Mjaaland S. Unexpected vaginal bleeding and COVID-19 vaccination in nonmenstruating women. Sci Adv. (2023) 9:adg1391. 10.1126/sciadv.adg1391 37738335 PMC10516485

[7] Ahmed SH Waseem S Shaikh TG Qadir NA Siddiqui SA Ullah I. SARS-CoV-2 vaccine-associated-tinnitus: a review. Ann Med Surg. (2022) 75:103293. 10.1016/j.amsu.2022.103293 PMC8788157 35096388

[8] Bolze A Mogensen TH Zhang SY Abel L Andreakos E Arkin LM. Decoding the human genetic and immunological basis of COVID-19 mRNA vaccine-induced myocarditis. J Clin Immunol. (2022) 42:1354–9. 10.1007/s10875-022-01372-9 36207567 PMC9546418

[9] Nishibayashi H Kishimoto S Sekiguchi K Okada S Ihara K. Myocarditis in 13-year-old monochorionic diamniotic twins after COVID-19 vaccination. J Clin Immunol. (2022) 42:1351. 10.1007/s10875-022-01360-z 36008643 PMC9410742

[10] Whitaker JA Ovsyannikova IG Poland GA. Adversomics: a new paradigm for vaccine safety and design vaccine adverse events and reactions HHS public access. Expert Rev Vaccines. (2015) 14:935–47. 10.1586/14760584.2015.1038249 25937189 PMC4630804

[11] Jaiswal S Ebert BL. Clonal hematopoiesis in human aging and disease. Science. (2019) 366:aan4673. 10.1126/science.aan4673 31672865 PMC8050831

[12] Agustin M Trifitriana M Danarti R. COVID arm as a common cutaneous manifestation after mRNA-1273 vaccination: a systematic review. BMC Infect Dis. (2023) 23:1–11. 10.1186/a12879-022-07973-4 36609222 PMC9817307

[13] Özütemiz C Krystosek LA Church AL Chauhan A Ellermann JM Domingo-Musibay E. Lymphadenopathy in COVID-19 vaccine recipients: diagnostic dilemma in oncologic patients. Radiology. (2021) 300:E290–4. 10.1148/radiol.2021210275 33625300 PMC7909072

[14] Ho TC Shen DHY Chang CC Chan HP Chuang KP Yuan CH. Immune response related to lymphadenopathy post COVID-19 vaccination. Vaccines. (2023) 11:30696. 10.3390/vaccines11030696 36992280 PMC10054375

[15] Verbeke R Hogan MJ Loré K Pardi N. Innate immune mechanisms of mRNA vaccines. Immunity. (2022) 55:1993–2005. 10.1016/j.immuni.2022.10.014 36351374 PMC9641982

[16] Whiteside SK Snook JP Williams MA Weis JJ. Bystander T cells: a balancing act of friends and foes. Trends Immunol. (2018) 39:1021. 10.1016/j.it.2018.10.003 30413351 PMC6269193

[17] He Y Hui Y Liu H Wu Y Sang H Liu F. Adult-onset familial hemophagocytic lymphohistiocytosis presenting with annular erythema following COVID-19 vaccination. Vaccines. (2022) 10:1436. 10.3390/vaccines10091436 36146514 PMC9501607

[18] Chinnici A Beneforti L Pegoraro F Trambusti I Tondo A Favre C. Approaching hemophagocytic lymphohistiocytosis. Front Immunol. (2023) 14:1210041. 10.3389/fimmu.2023.1210041 37426667 PMC10324660

[19] Win Z Weiner J Listanco A Patel N Sharma R Greenwood A. Systematic 18F-FDG- and 11C- evaluation of kinetics and distribution of muscle and lymph node activation measured by PBR28-PET/CT imaging, and whole blood and muscle transcriptomics after immunization of healthy humans with adjuvanted and unadjuvanted vaccines. Front Immunol. (2021) 11:613496. 10.3389/fimmu.2020.613496 33613536 PMC7893084

[20] Korzun T Moses AS Diba P Sattler AL Taratula OR Sahay G. From bench to bedside: implications of lipid nanoparticle carrier reactogenicity for advancing nucleic acid therapeutics. Pharmaceuticals. (2023) 16:1088. 10.3390/ph16081088 37631003 PMC10459564

